# High-Intensity Interval Training Interventions in Children and Adolescents: A Systematic Review

**DOI:** 10.1007/s40279-017-0753-8

**Published:** 2017-06-22

**Authors:** William T. B. Eddolls, Melitta A. McNarry, Gareth Stratton, Charles O. N. Winn, Kelly A. Mackintosh

**Affiliations:** 1 Applied Sports Science Technology and Medicine Research Centre (A-STEM), College of Engineering, Swansea University, A101 Engineering East, Bay Campus, Fabian Way, Swansea, SA1 8EN UK; 2 School of Sport Health and Exercise Science, University of Western Australia, Perth, WA Australia

## Abstract

**Background:**

Whilst there is increasing interest in the efficacy of high-intensity interval training in children and adolescents as a time-effective method of eliciting health benefits, there remains little consensus within the literature regarding the most effective means for delivering a high-intensity interval training intervention. Given the global health issues surrounding childhood obesity and associated health implications, the identification of effective intervention strategies is imperative.

**Objectives:**

The aim of this review was to examine high-intensity interval training as a means of influencing key health parameters and to elucidate the most effective high-intensity interval training protocol.

**Methods:**

Studies were included if they: (1) studied healthy children and/or adolescents (aged 5–18 years); (2) prescribed an intervention that was deemed high intensity; and (3) reported health-related outcome measures.

**Results:**

A total of 2092 studies were initially retrieved from four databases. Studies that were deemed to meet the criteria were downloaded in their entirety and independently assessed for relevance by two authors using the pre-determined criteria. From this, 13 studies were deemed suitable. This review found that high-intensity interval training in children and adolescents is a time-effective method of improving cardiovascular disease biomarkers, but evidence regarding other health-related measures is more equivocal. Running-based sessions, at an intensity of >90% heart rate maximum/100–130% maximal aerobic velocity, two to three times a week and with a minimum intervention duration of 7 weeks, elicit the greatest improvements in participant health.

**Conclusion:**

While high-intensity interval training improves cardiovascular disease biomarkers, and the evidence supports the effectiveness of running-based sessions, as outlined above, further recommendations as to optimal exercise duration and rest intervals remain ambiguous owing to the paucity of literature and the methodological limitations of studies presently available.

## Key Points

**Table:** 

High-intensity interval training can improve certain cardiovascular health parameters in children and adolescents.
Evidence supporting the overall effectiveness of high-intensity interval training as a means of eliciting improvements to other health outcomes, specifically body composition and blood pressure, remains unclear.
While this review enables the establishment of suggested guidelines for high-intensity interval training protocols, recommendations for some protocol details remain unclear.

## Introduction

Worldwide, the prevalence of childhood and adolescent obesity has reached unparalleled levels [1, 2]. Specifically, in the UK, approximately 28% of children are classified as overweight or obese [2, 3], representing a significant burden on health services. Indeed, current estimates suggest that obesity and its deleterious health consequences, such as type 2 diabetes mellitus [4] and coronary heart disease [5], cost the National Health Service £5.1 billion per year [6]. Whilst some reports suggest a plateau in paediatric obesity over the last decade [7], others suggest that UK obesity levels have increased between 1980 and 2014 by 48 and 39% in boys and girls, respectively [2]. Of concern, paediatric obesity has been associated with an increased prevalence of cardiometabolic risk factors [8], which have been shown to track into later life [9] and increase the risk of premature mortality [9, 10]. Although the causes of obesity and cardiometabolic risk are multifaceted, low physical activity levels, as well as high engagement in sedentary pursuits, have been identified as key contributory factors [9, 11–13]. Specifically, according to the latest statistics, in England, only 21% of boys and 16% of girls meet UK physical activity guidelines of at least 60 min of moderate-to-vigorous physical activity every day [3], with physical activity levels further declining with age [14–16]. Effective interventions targeted at increasing youth physical activity levels are therefore imperative.

While traditional interventions designed to increase physical activity and improve health have principally used moderate-intensity continuous exercise [17–19], the relevance of such programmes to the sporadic high-intensity nature of children’s habitual play patterns has been questioned [20]. Consequently, high-intensity interval-based programmes have recently been investigated as a potentially potent and time-efficient form of physical activity and health promotion [21]. Indeed, whilst exercise, a sub-component of physical activity, is structured and conducted for the health-associated benefits, it could provide a necessary mediatory step to provoke positive long-term behavioural change. However, to date, studies have demonstrated varied success at eliciting significant improvements [22–25], potentially owing to a lack of consensus regarding an optimum high-intensity interval training (HIIT) intervention protocol with regard to exercise intensity, frequency and duration. Nonetheless, recent systematic reviews [26, 27] highlighted that HIIT can elicit greater improvements in health-related parameters (i.e. cardiometabolic health and body composition) in adolescents, compared with traditional programmes [23, 28–30]. However, no systematic reviews have investigated the effects of HIIT on primary school-aged children, the identified potential differences between pre-pubertal and pubertal youth in the adaptations elicited or provided optimal HIIT protocol recommendations. Therefore, the purpose of this review was to systematically synthesise the scientific literature regarding HIIT on improving body composition, cardiometabolic health and cardiovascular health in children and adolescents and to establish an optimal HIIT protocol with regard to session structure, intensity, frequency and duration.

## Methodology

In line with the Preferred Reporting Items for Systematic Reviews and Meta-Analyses [31], the following methodology details the review’s inclusion criteria, search strategy, data collection and study analysis protocols.

### Inclusion Criteria

#### Types of Study

The present review included studies involving interventions targeted at reducing obesity-related physiological parameters with a principal focus on one of the following: high-intensity physical activity, high-intensity exercise/training or high-intensity intermittent/interval exercise/training interventions.

#### Types of Participant

Studies incorporating children and adolescents between the ages of 5 and 18 years were included. These age constraints were applied to all children and adolescents without disability, irrespective of whether they were a healthy weight, overweight or obese. Studies that used physical activity interventions as part of a treatment for specific illnesses were excluded.

#### Intervention Variables and Outcome Measures

To be included in the review, studies were required to report a minimum of one intervention exercise session intensity variable and one outcome measure, measured at baseline and post-intervention and compared with either a moderate-intensity exercise intervention or control group.

#### Intervention Intensity Variables

Interventions were defined as high intensity if: (1) the intensity was ≥90% peak oxygen uptake [32]; (2) had an intensity that was ≥100% maximal aerobic speed [33]; and/or (3) ensured that the participant’s heart rate was ≥90% of their peak heart rate [34, 35]. There were no restrictions applied regarding the duration of the intervention.

#### Primary Outcomes

Primary outcomes included cardiometabolic health markers, namely mean systolic (SBP) and diastolic (DBP) blood pressure, body composition in the form of body mass index (BMI), body fat percentage (BF%) and fat-free mas, and cardiovascular disease (CVD) biomarker analysis including at least one of the following: glucose, insulin, triglyceride and total cholesterol, as well as its sub-fractions, low-density lipoprotein cholesterol and high-density lipoprotein cholesterol.

### Search Strategy

Electronic databases were searched until September 2016, with no restriction set on the publication year. The PubMed and SCOPUS databases were explored using the following keyword search strategy, devised by the research team and verified by a subject librarian: (high intensity training OR high intensity exercise* OR high intensity activit* OR high intensity intermittent training OR intensity intermittent exercise* OR high intensity intermittent activit* or high intensity interval training OR high intensity interval exercise* OR high intensity interval activit*) AND (child* OR children OR pediatric OR paediatric OR adolescen* OR juvenile*) AND (health OR healthy). Inclusion of at least one of the keywords was required in the study title for it to be considered. Studies were excluded based on language; only studies written in English were included. Additional studies were identified by searching the reference lists of included studies. Google Scholar and ResearchGate were also searched to identify studies that were potentially overlooked by the database searches.

### Data Collection and Analysis

Pertinent study abstracts from the stated search strategies were downloaded and independently screened. Studies that were deemed to meet the criteria were downloaded in their entirety and independently assessed for relevance by two authors using the pre-determined criteria. When study information was missing, the research team attempted to contact the primary author of the incomplete study. If the author failed to respond, the study was excluded.

### Effect Size

Cohen’s d was used to determine the standardised mean effect of HIIT on the previously outlined health-related outcome measures compared with a control group or a moderate- or light-intensity group [36]. Confidence intervals (CIs) were calculated by applying an equation recommended by Nakagawa and Cuthill [37], employing standard error calculations [38]. For studies that provided values for both moderate and control groups, moderate group values were included as the comparison. Additionally, effect size was not calculated for studies that failed to disclose post-intervention mean values.

### Risk of Bias Assessment

Risk of bias was assessed independently by two reviewers using the Cochrane risk of bias tool (RoB 2.0) [39]. Using the RoB 2.0 tool, studies were awarded an overall risk of bias grade of either high, some or low risk of bias. This overall grade was calculated by assessing five domains: (A) bias arising from the randomisation process; (B) bias owing to deviations from intended interventions; (C) bias owing to missing outcome data; (D) bias in measurement of the outcome; and (E) bias in selection of the reported result.

### Heterogeneity Assessment

Because of the variation of the study characteristics in this review, for example between interventions, outcome measures and cohort populations, it was deemed unsuitable to amalgamate the results for a meta-analysis. Therefore, the results in this review were analysed narratively.

## Results

**Fig. 1:**
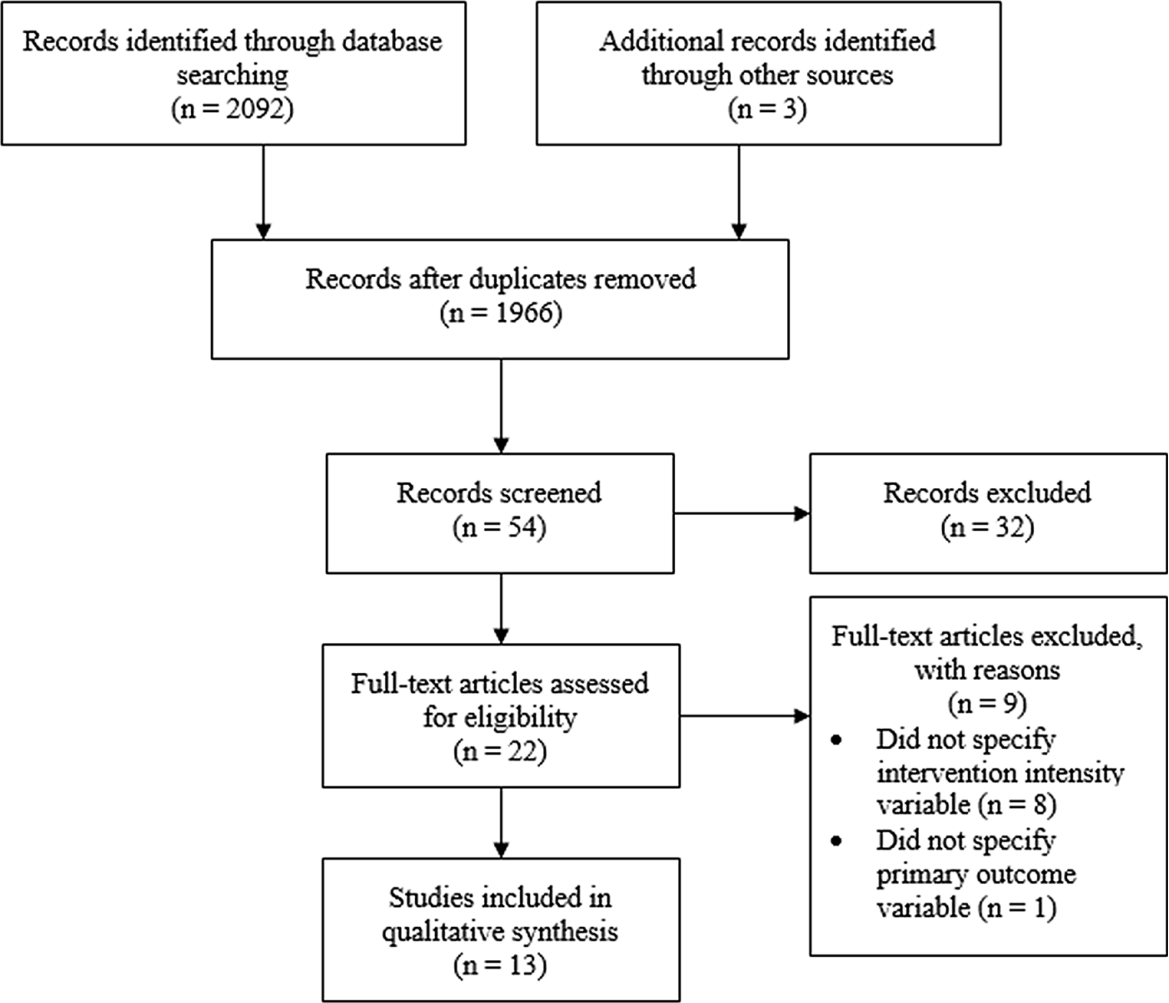
Phases of study selection during data collection

The database search generated 2092 studies. Once duplicates were removed, 54 title/abstracts were screened for eligibility, with the reference list search producing three further studies. From this, 13 studies were deemed suitable. The screening process is shown in Fig. 1. In total, 7 of the 13 studies included in this review examined the effects of HIIT interventions on pre-pubertal participants, the characteristics of which are summarised in Table 1, with the remaining six studies examining the effects of HIIT interventions on pubertal participants (Table 2).

**Table 1:** Characteristics of studies examining pre-pubertal participants

References	Sample population	Maturation	INT duration (wk)	INT type	Group size (n)	Modality/ intensity	Repeated bouts/ frequency	Exercise bout/ recovery duration	Protocol duration (including recovery)	Total exercise INT duration
Baquet et al. [22]	Pre-pubertal primary school children; N = 53 (23 boys; 8–11 years)	Maturation measured, but not reported	7	HIIT protocol	33	Shuttle runs (100–130 % MAS)	Bouts: 5–10 Sets: 1–4 3-min rest between set (2 times weekly)	10–20 s/ 10–20 s	30 min	7 h
				Control	20					
Baquet et al. [40]	Primary school children; N = 77 (43 boys; 9.6 ± 1.0 years)	Stage 1 = 40 boys, 29 girls Stage 3 = 3 boys, 5 girls combined <3	7	HIIT protocol	22	Shuttle runs (100–130 % MAS)	Bouts: 5–10 Sets: 1–4 3-min rest between set (3 times weekly)	10–30 s/ 10–30 s	25–35 min	10 h and 30 min
				Moderate	22	Shuttle runs (80–85 % MAS)	Bouts: 1–4 (3 times weekly)	6–18 min/ 5 min	18–39 min	7 h and 21 min
				Control	19					
Baquet et al. [41]	Pre-pubertal children; N = 100 (46 boys; 9.7 ± 0.8 years)	Stage 1 = 46 boys, 25 girls Stage 3 = 29 girls combined <3	7	HIIT protocol	47	Shuttle runs (100–130 % MAS)	Bouts: 5–10 Sets: 1–4 3-min rest between set (2 times weekly)	10–20 s/ 10–20 s	30 min	7 h
				Control	53					
Lambrick et al. [42]	Obese and normal weight children; N = 55 (32 boys)	Peak height velocity at baseline: INT = 2.7 ± 0.1 CON = 2.65 ± 0.05	6	HIIT protocol	28	Child specific games (93% mean HR_max_)	Bouts: 7 (2 times weekly)	6 min/2 min	60 min	12 h
				Control	27					
Lau et al. [24]	Overweight primary school children; N = 48 (36 boys; 10.4 ± 0.9 years)	Maturation not reported	6	HIIT protocol	15	Shuttle runs (120 % MAS)	Bouts: 12 (3 times weekly)	15 s/15 s	6 min	1 h and 48 min
				LIT protocol	21	Shuttle runs (100 % MAS)	Bouts: 16 (3 times weekly)	15 s/15 s	8 min	2 h and 24 min
				Control	12					
Nourry et al. [43]	Pre-pubertal children; N = 18 (11 boys; 10.0 ± 0.8 years)	Stage 1 = 18	8	HIIT protocol	9	Shuttle runs (100–130 % MAS)	Bouts: 10 Sets: 4 times (2 times weekly)	10–20 s/ 10–20 s	30 min	8 h
				Control	9					
Rosenkranz et al. [28]	Pre-pubertal children; N = 16 (2 boys; 7–12 years)	Stage 1 = 16	8	HIIT protocol	8	Shuttle runs (100–130 % MAS)	Bouts: 5–10 Sets: 4 times (2 times weekly)	10–20 s/ 10–20 s	30 min	8 h
				Control	8					

*HIIT* high-intensity interval training, *HR*_*max*_ heart rate maximum, *INT* intervention, *LIT* light-intensity training, *MAS* maximal aerobic speed

**Table 2:** Characteristics of studies examining pubertal participants

References	Sample population	Maturation	INT duration (wk)	INT type	Group size (n)	Modality/ intensity	Repeated bouts/ frequency	Exercise bout/ recovery duration	Protocol duration (including recovery)	Total exercise INT duration
Baquet et al. [25]	Secondary school children; N = 551 (290 boys; 11–16 years)	Not reported	10	HIIT protocol	503	Shuttle runs (100–120 % MAS)	Bouts: 10 Sets: 3 3-min rest between set (1 times weekly)	10 s/10 s	60 min	10 h
				Control	48					
Boddy et al. [44]	Secondary school girls; N = 16 (11.8 ± 0.3 years)	Peak height velocity at baseline:	3	HIIT protocol	8	Dance class (>93.4 mean % HR_max_)	Bouts: 6 (4 times weekly)	30 s/45 s	20 min	4 h
		INT = 0.187 ± 0.37 CON = 0.028 ± 0.427		Control	8					
Racil et al. [30]	Obese female adolescents; N = 47 (14.2 ± 1.2 years)	Not reported	12	HIIT protocol	17	Various types of interval training (100% MAS)	Bouts: 8–16 (3 times weekly)	15 s/15 s	4–8 min	3 h and 36 min
				Moderate	16	Various types of interval training (80% MAS)	Bouts: 8–16 (3 times weekly)	15 s/15 s	4–8 min	3 h and 36 min
				Control	14					
Sperlich et al. [45]	Male soccer players; N = 19 (13.5 ± 0.4 years)	Maturation not reported	5	HIIT protocol	9	Various types of interval training (90–95 % HR_max_)	Bouts: 4–12 (3–4 times weekly)	30 s to 4 min/30 s to 3 min	30 min	8 h and 45 min
				Moderate	8	Various types of interval training (50–70 % HR_max_)	Bouts: 1–5 (3–4 times weekly)	10–30 min/1–3 min	40–60 min	14 h and 35 min
Tjønna et al. [23]	Overweight and obese adolescents; N = 54 (26 boys; 14.0 ± 0.3 years)	Maturation not reported	12	HIIT protocol	28	Treadmill walking/running (90–95 % HR_max_)	Bouts: 4 (2 times weekly)	4 min/3 min	25 min	10 h
				Control	26					
Weston et al. [29]	Adolescent secondary school children; N = 101 (62 boys; 14.0 ± 0.3 years)	Peak height velocity at baseline:	10	HIIT protocol	41	Games based (>90% mean HR_max_)	Bouts: 4–7 (3 times weekly)	45 s/90 s	7 min 30 s to 14 min 15 s	5 h and 30 min
		INT = 0.3 ± 1.0 Control = 0.5 ± 1.3		Control	60					

*HIIT* high-intensity interval training, *HR*_*max*_ heart rate maximum, *INT* intervention, *MAS* maximal aerobic speed

### Risk of Bias

The methodological rigour of studies included in this review, according to the risk of bias assessment, is presented in Table 3. Seven studies were considered to have a high risk of bias [22, 24, 25, 29, 40, 41, 45], whereas only two [28, 43] and four [23, 30, 42, 44] studies were considered to have some or low risk of bias, respectively. In studies deemed to have a high or some risk of bias [22, 24, 25, 28, 29, 40, 41, 43, 45], the bias arose from the randomisation process (domain A).

**Table 3:** Risk of bias assessment

References	Domain A	Domain B	Domain C	Domain D	Domain E	Total
Baquet et al. [22]	High	Low	Low	Low	Low	High
Baquet et al. [25]	High	Low	Low	Low	Low	High
Baquet et al. [40]	High	Low	Low	Low	Low	High
Baquet et al. [41]	High	Low	Low	Low	Low	High
Boddy et al. [44]	Low	Low	Low	Low	Low	Low
Lambrick et al. [42]	Low	Low	Low	Low	Low	Low
Lau et al. [24]	High	Low	Low	Low	Low	High
Nourry et al. [43]	Some	Low	Low	Low	Low	Some
Racil et al. [30]	Low	Low	Low	Low	Low	Low
Rosenkranz et al. [28]	Some	Low	Low	Low	Low	Some
Sperlich et al. [45]	High	Low	Low	Low	Low	High
Tjønna et al. [23]	Low	Low	Low	Low	Low	Low
Weston et al. [29]	High	Low	Low	Low	Low	High

Domain: (A) bias arising from the randomisation process, (B) bias owing to deviations from intended interventions, (C) bias owing to missing outcome data, (D) bias in measurement of the outcome, (E) bias in selection of the reported result. Total overall risk of bias grade was calculated by assessing the five domains [A–E]

### Body Mass and Composition

All 13 included studies reported the effect of HIIT compared with moderate-intensity exercise or a control group on BMI (*n* = 9), BF% (*n* = 9) or fat-free mass (*n* = 1). The results revealed little evidence to suggest that HIIT can elicit significant changes in body composition (Table 4), although Tjønna et al. [23] and Racil et al. [30], both of which were assessed to be at low risk of bias, reported significant improvements in BMI and BF% associated with a medium-to-large effect sizes following a 3-month intervention.

**Table 4:** Baseline and post-intervention changes to body mass/composition and effect size between high-intensity interval training (HIIT) and control/moderate protocols

References	Outcome measure	HIIT (mean change from baseline)	Control/moderate (mean change from baseline)	Effect size (Cohen’s *d*)	95% CI
Baquet et al. [25]	BMI (kg/m^2^)	0.40**	0.60**	**−**0.14	**−**0.78 to 0.49
Baquet et al. [40]	BMI (kg/m^2^)	0.10	**−**0.30/0.20	**−**0.10	**−**0.78 to 0.58
Boddy et al. [44]	BMI (kg/m^2^)	**−**0.50	0.20	0.93	0.30 to 1.56
Lambrick et al. [42]	BMI (kg/m^2^)	0.00	0.00	0.23	**−**0.40 to 0.87
Lau et at. [24]	BMI (kg/m^2^)	0.20	0.10/0.40*	0.42	**−**0.18 to 1.01
Racil et al. [30]	BMI (kg/m^2^)	**−**3.20*	0.30/**−**1.70*	**−**1.41	**−**3.52 to 0.69
Rosenkranz et al. [28]	BMI (kg/m^2^)	**−**1.40	0.00	**−**0.06	**−**0.73 to 0.61
Tjønna et al. [23]	BMI (kg/m^2^)	**−**0.70**	**−**0.20	**−**1.50	**−**2.06 to **−**0.94
Weston et al. [29]	BMI (kg/m^2^)	**−**0.60^a^	0.80^a^	N/A	N/A
Baquet et al. [25]	Body fat (%)	1.60*	1.30*	**−**0.31	**−**0.94 to 0.33
Baquet et al. [22]	Body fat (%)	**−**0.90	**−**0.70	0.10	**−**0.51 to 0.71
Baquet et al. [41]	Body fat (%)	0.10	0.10	0.02	**−**0.66 to 0.69
Boddy et al. [44]	Body fat (%)	0.22	0.46	0.61	0.14 to 1.07
Lambrick et al. [42]	Body fat (%)	**−**0.10	0.40	0.08	**−**0.49 to 0.65
Nourry et al. [43]	Body fat (%)	1.50	0.50	0.14	**−**0.56 to 0.84
Racil et al. [30]	Body fat (%)	**−**3.90*	**−**0.50/**−**3.40*	**−**0.59	**−**1.06 to **−**0.12
Rosenkranz et al. [28]	Body fat (%)	**−**2.20	**−**1.00	**−**0.17	**−**0.80 to 0.45
Tjønna et al. [23]	Body fat (%)	**−**0.90**	**−**0.30	3.00	2.35 to 3.65
Sperlich et al. [45]	FFM (kg)	1.00	0.90	0.71	0.28 to 1.13

*BMI* body mass index, *CI* confidence interval, *FFM* fat-free mass, *N/A* effect size not calculated because of no reported post-intervention means

* *p* **<** 0.05, ** *p* **<** 0.01, significantly different from baseline

^a^Values adjusted for sex, baseline value and maturity offset

It is pertinent to note that whilst other studies failed to find a significant improvement in measures of body mass or composition, there was a general trend for a greater change in body mass and composition in the HIIT group [22, 28, 29, 43, 45]. The exception to this was Baquet et al. [25], who saw significant increases to BMI and BF% for both the HIIT protocol and the control group; however, this study was deemed to have a high risk of bias. While no significant benefits were reported in pre-pubertal children, significant improvements in body mass and composition have been demonstrated in pubertal children [23, 30], suggesting a potential maturational effect. There were insufficient data to examine potential sex differences regarding the efficacy of HIIT in eliciting significant changes in body composition.

### Cardiovascular Health

#### Blood Pressure

Five studies investigated the effect of HIIT on SBP and DBP, with the majority concluding significant benefits were obtained (Table 5). Specifically, two studies that were assessed to be of low risk of bias, Tjønna et al. [23] and Racil et al. [30], reported significant improvements in both SBP and DBP following the intervention, although it is pertinent to note the low effect sizes associated with the improvements reported for SBP and DBP in Racil et al. [30] (Table 5). Whilst the remaining studies [28, 29, 44] reported no significant differences, they demonstrated a trend towards a lower SBP and DBP. Interestingly, Boddy et al. [44], demonstrating methodological rigour through a low risk of bias, found an increase in both DBP and SBP in the HIIT group and reductions in the control group, though not significant. Regarding maturation differences, no studies examining pre-pubertal children reported significant changes, whereas significant improvements were reported in pubertal populations [23, 30]. Sex differences could not be investigated because of insufficient data.

**Table 5:** Baseline to post-intervention changes in systolic blood pressure/diastolic blood pressure (SBP/DBP) and effect size between high-intensity interval training (HIIT) and control/moderate protocols

References	Outcome measure (mmHg)	HIIT (mean change from baseline)	Control/moderate (mean change from baseline)	Effect size (Cohen’s *d*)	95% CI
Boddy et al. [44]	SBP	10.10	−1.40	0.34	0.08 to 0.61
Racil et al. [30]	SBP	−0.60*	0.00/−0.40*	0.00	−0.26 to 0.26
Rosenkranz et al. [28]	SBP	−2.20	−2.50	−0.54	−0.82 to −0.26
Tjønna et al. [23]	SBP	−9.40**	−2.50*	−2.00	−2.31 to −1.69
Weston et al. [29]	SBP	−5.00^a^	−1.00^a^	N/A	N/A
Boddy et al. [44]	DBP	5.90	−4.10	1.14	0.75 to 1.52
Racil et al. [30]	DBP	−6.00*	−1.00/−4.00*	−0.32	**−**0.64 to 0.01
Rosenkranz et al. [28]	DBP	−2.50	−1.70	−0.83	−1.18 to −0.48
Tjønna et al. [23]	DBP	−5.50**	1.80	−1.50	−1.89 to −1.11
Weston et al. [29]	DBP	−6.00^a^	−4.00^a^	N/A	N/A

*CI* confidence interval, *N/A* effect size not calculated because of no reported post-intervention means

* *p* < 0.05; ** *p* < 0.01 significantly different from baseline

^a^Values adjusted for sex, baseline value and maturity offset

#### Cardiovascular Disease Biomarker Health

Four studies examined the effect of HIIT on CVD biomarkers, specifically blood glucose (*n* = 4), total cholesterol (*n* = 2), high-density lipoprotein cholesterol (*n* = 3), low-density lipoprotein cholesterol (*n* = 1), blood triglycerides (*n* = 3) and insulin (*n* = 2). The results, in Table 6, support HIIT as an effective strategy for improving CVD biomarker health. All of the studies demonstrated significant [23, 28, 30] or clinically substantial [29] improvements in multiple CVD biomarker outcome measures. Tjønna et al. [23] reported greater significant improvements for blood glucose in favour of the HIIT group (*p* < 0.01) compared with the control group (*p* < 0.05), with an effect size indicating large clinically important differences (*d* = −1.43, 95% CI −3.01 to 0.16). Additionally, results from Rosenkranz et al. [28] suggest large significant reductions in total cholesterol compared with the control group. Further, the associated effect size (*d* = −0.93, 95% CI −1.16 to −0.70) suggested highly clinically important differences. Rosenkranz et al. [28] saw significant reductions (*p* < 0.05) in low-density lipoprotein cholesterol as a result of HIIT, with a large and clinically important difference (*d* = −1.67, 95% CI −2.03 to −1.31) compared with a small non-significant increase in the control group. Examining blood insulin results in Tjønna et al. [23], larger significant reductions in the HIIT group were found compared with the control group, with further significant reductions after a 12-month follow-up. In addition, high-density lipoprotein cholesterol significantly improved in the HIIT group compared with a non-significant increase in the control group. Furthermore, Racil et al. [30] reported significant reductions in blood glucose and insulin in both HIIT and moderate-intensity protocols compared with a control group, with a greater improvement reported in the HIIT group for both measures. Effect sizes for both blood glucose and insulin were low to moderate (*d* = 0.32, 95% CI −0.44 to 0.13) and large (*d* = −0.82, 95% CI −1.55 to −0.10), respectively.

**Table 6:** Baseline and post-intervention changes in cardiovascular disease biomarkers and effect size between high-intensity interval training (HIIT) and control/moderate protocols

References	Outcome measure	HIIT (mean change from baseline)	Control/moderate (mean change from baseline)	Effect size (Cohen’s *d*)	95% CI
Racil et al. [30]	Glucose (mmol∙L^−1^)	−0.20*	0.00/−0.20*	−0.32	−0.44 to 0.13
Rosenkranz et al. [28]	Glucose (mg/dL)	5.20	0.40	−0.16	−1.80 to 1.17
Tjønna et al. [23]	Glucose (mmol∙L^−1^)	−0.30**	−0.10	−1.43	−3.01 to 0.16
Weston et al. [29]	Glucose (mmol∙L^−1^)	−0.10^a^	−0.03^a^	N/A	N/A
Rosenkranz et al. [28]	Total cholesterol (mg/dL)	−22.00*	2.40	−0.93	−1.16 to −0.70
Weston et al. [29]	Total cholesterol (mmol∙L^−1^)	−0.24^a^	0.00^a^	N/A	N/A
Rosenkranz et al. [28]	HDL-cholesterol (mg/dL)	9.90	3.60	0.42	0.02 to 0.81
Tjønna et al. [23]	HDL-cholesterol (mmol∙L^−1^)	0.11*	0.09	0.35	−0.50 to 1.20
Weston et al. [29]	HDL-cholesterol (mmol∙L^−1^)	−0.14^a^	−0.24^a^	N/A	N/A
Rosenkranz et al. [28]	LDL-cholesterol (mg/dL)	−34.80*	−5.60	−1.67	−2.03 to −1.31
Rosenkranz et al. (2012) [28]	Triglycerides (mg/dL)	23.50	3.50	−0.07	−0.33 to 0.19
Tjønna et al. [23]	Triglycerides (mmol∙L^−1^)	−0.50	−0.10	−0.71	−1.57 to 0.14
Weston et al. [29]	Triglycerides (mmol∙L^−1^)	−0.05^a^	0.18^a^	N/A	N/A
Racil et al. [30]	Insulin (IU mL^−1^)	−5.70*	−0.80/−4.30*	−0.82	−1.55 to −0.10
Tjønna et al. [23]	Insulin (pmol/L)	−54.30*	−33.00*	−0.46	−0.70 to −0.22

*CI* confidence interval, *HDL* high-density lipoprotein cholesterol, *LDL* low-density lipoprotein cholesterol, *N/A* effect size not calculated because of reported post-intervention means

* *p* < 0.05; ** *p* < 0.01 significantly different from baseline

^a^Values adjusted for sex, baseline value and maturity offset

Notwithstanding the limited improvement in blood triglycerides, Weston et al. [29] reported clinically substantial beneficial effects as a result of HIIT despite increased triglyceride levels reported in the control group. Despite these encouraging findings, it is pertinent to note that the studies by Rosenkranz et al. [28] and Weston et al. [29] were assessed to have some and a high risk of bias, respectively; therefore, caution should be taken when interpreting these studies as methodological limitations may have confounded the results. There was no effect owing to maturation on CVD biomarkers, with significant or clinically substantial improvements found in both pre-pubertal [28] and pubertal [23, 29, 30] children. Sex differences were not reported and, therefore, their effect is unknown.

### High-Intensity Interval Training Intervention Protocol

All studies included in this review provided a detailed description of their intervention protocol in terms of session structure, duration, intensity and frequency, in addition to intervention duration; key details of these are summarised in Tables 1 and 2. Based on the four studies that demonstrated significant health improvements [23, 28–30], a running-based HIIT intervention at an intensity of >90% heart rate maximum/100–130 % maximal aerobic velocity, two to three times a week with a minimum intervention duration lasting 7 weeks could be considered the suggested practice. However, suggested exercise session duration and rest intervals remain ambiguous owing to the variance across the studies, a notion also supported by Baquet et al. [46].

## Discussion

The aim of the current review was to synthesise previous literature that examined HIIT in children and adolescents and establish its potential effect on body composition, cardiometabolic health and cardiovascular health. In addition, this review aimed to identify an optimal HIIT protocol with regard to session structure, intensity, frequency and duration. In accord with this aim, 13 studies were evaluated providing evidence suggesting that HIIT can significantly improve certain health parameters in children and adolescents. However, evidence supporting the overall effectiveness of HIIT as a means of eliciting improvements to all the specified health outcomes remains unclear. Some guidelines for a HIIT protocol were established, though recommendations for certain protocol details remain unidentified.

Advancing previous reviews [26, 27], the findings of the current review suggest that pubertal children may achieve a greater benefit as a result of HIIT when compared with pre-pubertal children, a topic that has been widely debated [47–50]. However, it is pertinent to note that this may be a consequence of several methodological factors that limit the interpretation of previous studies. First, the duration of the HIIT interventions examining pubertal participants tended to be longer than those in pre-pubertal children. Given the present findings suggesting that a minimum of 7 weeks is required for significant adaptations to be manifest, this shorter intervention duration may lead to erroneous conclusions regarding the efficacy of HIIT in this population.

Furthermore, differences in the participant characteristics between pre-pubertal and pubertal studies with regard to baseline body mass or body composition and health status may confound interpretation of inter-study differences and their attribution to maturity per se. Specifically, the majority of studies in pre-pubertal children used those of a normal weight compared with the inclusion of overweight or obese participants in pubertal studies, which may predispose these latter studies to demonstrating greater health benefits, irrespective of their biological age. Moreover, both Tjønna et al. [23] and Racil et al. [30] did not report maturation stages, subsequently casting ambiguity over the cohort’s true maturational stage. Despite this, when focusing on the additional study involving pubertal children [29], it generally elicited greater improvements in outcome measures when compared with the study involving pre-pubertal children [28]. Moreover, only two of the studies involving pubertal children that demonstrated positive significant results [23, 30] considered dietary intake; a failure to account for changes in dietary intake, which is strongly associated with cardiometabolic health [51] and obesity [52] in children and adolescents, confounds the interpretation of the results and their attribution to the exercise per se. Finally, in addition to the aforementioned methodological limitations, the interpretation of the overall findings of this study may also be limited by a mixed risk of bias between the significant studies.

An additional factor that may contribute to the collective ambiguity regarding the overall effectiveness of HIIT is the ‘compensation effect’. Specifically, a recent study has suggested that school children appear to compensate for increased physical activity levels, with a reduction in physical activity undertaken the following day [53]. Additionally, the ‘activitystat’ hypothesis suggests that increased levels of physical activity during one part of the day may result in a compensatory decrease in physical activity in another part [54]. Therefore, there is scope to suggest that prescribing exercise that reflects the characteristics of children’s comparatively elevated levels of habitual play [55] may result in a decrease in habitual physical activity levels that day or the subsequent day. This therefore highlights the need to measure habitual physical activity, as was the case for only four of the studies included within this review [23, 28, 29, 44], alongside the previously outlined study outcomes. Regarding the effect of HIIT between sexes, no conclusions could be drawn as none of the studies included in this review provided a breakdown of between sex differences. This could have implications for future research, given the possibility that effects of exercise interventions on body size [56, 57], cardiorespiratory health [58] and cardiometabolic health [57] may be sex dependent in children. Therefore, future studies should endeavour to report a more expansive breakdown of results, thus providing clarification as to possible sex and maturational differences associated with HIIT.

Whilst the studies within this review have advanced our understanding regarding the influence of HIIT in children under laboratory-based conditions, the relatively small sample sizes and intervention delivery methods highlight potential issues regarding larger scale implementation of HIIT. Schools have frequently been used as a foundation in the implementation of physical activity interventions [59, 60] because of their access to a greater population of children, who spend 40% of their waking hours there [61], and are widely accepted as one of the most effective locations to promote physical activity and health. Furthermore, previous studies have demonstrated how short-term HIIT interventions have been successfully embedded within the school timetable [44, 62]. It has been suggested that HIIT can allow for greater class control compared with conventional physical education lessons, and can be adapted to include specific movements related to different sports [63]. Therefore, given the promising findings regarding the effectiveness of HIIT, future research may wish to consider how long-term HIIT interventions could be incorporated within the school environment. A key consideration in the development of future interventions, and participants’ engagement in and adherence to the devised program, is a participant’s perceived enjoyment. Whilst HIIT has been suggested to be a preferable exercise modality to more conventional aerobic exercise [26], further research is required, with only one study in the present review considering this aspect of intervention development and implementation [42]. Finally, the sustained post-intervention efficacy of HIIT interventions that reported significant improvements to body composition and cardiometabolic and cardiovascular health in children and adolescents remains indeterminate owing to a predominant scarcity of studies reporting a post-intervention follow-up. Therefore, future studies should incorporate a follow-up period within their study design to assess the long-term post-intervention sustainability of positive HIIT elicited benefits.

## Conclusion

High-intensity interval training is a time-effective method of improving CVD biomarker health in children and adolescents. However, evidence supporting its effectiveness in additional health measures remains equivocal. This review suggests that running-based sessions, at an intensity of >90% maximum heart rate/100–130% maximal aerobic velocity, two to three times a week and with a minimum intervention duration lasting >7 weeks, elicit improvements in health markers; however, these findings are limited by the mixed risk of bias between the significant studies. Further recommendations as to exercise duration and rest intervals remain ambiguous owing to the paucity and methodological limitations of studies presently available.
